# Skew log-concavity of the Boros-Moll sequences

**DOI:** 10.1186/s13660-017-1394-z

**Published:** 2017-05-18

**Authors:** Eric H Liu

**Affiliations:** School of Business Information, Shanghai University of International Business and Economics, Shanghai, 201620 P.R. China

**Keywords:** 05A20, 05A10, log-concavity, skew log-concavity, the Boros-Moll sequence

## Abstract

Let $\{T(n,k)\}_{0\leq n < \infty, 0\leq k \leq n} $ be a triangular array of numbers. We say that $T(n,k)$ is skew log-concave if for any fixed *n*, the sequence $\{T(n+k,k)\}_{0 \leq k <\infty}$ is log-concave. In this paper, we show that the Boros-Moll sequences are almost skew log-concave.

## Introduction and main result

Boros and Moll [1, 2] explored a special class of Jacobi polynomials in their study of a quartic integral. They have shown that for any $a>-1$ and any nonnegative integer *m*,

$$ \int_{0}^{\infty}\frac{1}{(x^{4}+2ax^{2}+1)^{m+1}}\,dx = \frac{\pi}{2^{m+3/2}(a+1)^{m+1/2}}P_{m}(a), $$

where

1.1 $$ P_{m}(a)=\sum_{j,k} {2m+1 \choose 2j}{m-j \choose k}{2k+2j \choose k+j} \frac{(a+1)^{j}(a-1)^{k}}{2^{3(k+j)}}. $$

Using Ramanujan’s master theorem, Boros and Moll [2] derived the following formula for $P_{m}(a)$:

1.2 $$ P_{m}(a)=2^{-2m}\sum _{k}2^{k}{2m-2k \choose m-k} {m+k \choose k}(a+1)^{k}, $$

which implies that the coefficient of $a^{i}$ in $P_{m}(a)$ is positive for $0\leq i \leq m$. Let $d_{i}(m)$ be given by

1.3 $$ P_{m}(a)=\sum_{i=0}^{m}d_{i}(m)a^{i}. $$

The polynomial $P_{m}(a)$ is called the Boros-Moll polynomial, and the sequence $\{d_{i}(m)\}_{0 \leq i \leq m}$ of the coefficients is called a Boros-Moll sequence. From (1.3), we know that $d_{i}(m)$ can be given by

1.4 $$ d_{i}(m)=2^{-2m}\sum _{k=i}^{m}2^{k}{2m-2k \choose m-k} {m+k \choose k}{k\choose i}. $$

Some combinatorial properties of $\{d_{i}(m)\}_{0 \leq i \leq m}$ have been proved. Boros and Moll [1] proved that the sequence $\{d_{i}(m)\}_{0 \leq i \leq m}$ is unimodal, and the maximum element appears in the middle. Recall that a sequence $\{a_{i}\}_{0 \leq i \leq m}$ of real numbers is said to be unimodal if there exists an index $0 \leq j \leq m$ such that

$$a_{0}\leq a_{1}\leq\cdots\leq a_{j-1} \leq a_{j}\geq a_{j+1}\geq\cdots \geq a_{m} $$

and $\{a_{i}\}_{0 \leq i \leq m}$ is said to be log-concave if

1.5 $$ a_{i}^{2}-a_{i+1}a_{i-1} \geq0,\quad 1\leq i \leq m, $$

where $a_{-1}=a_{m+1}=0$. Moll [2] conjectured that the sequence $\{d_{i}(m)\}_{0 \leq i \leq m}$ is log-concave. Kauers and Paule [3] proved this conjecture based on recurrence relations found using a computer algebra approach. Recently, Chen and Xia [4] showed that the sequence $\{d_{i}(m)\}_{0 \leq i \leq m}$ satisfies the strongly ratio monotone property which implies the log-concavity and the spiral property. They [5] also confirmed a conjecture of Moll which says that $\{i(i+1) (d_{i}^{2}(m) -d_{i-1}(m)d_{i+1}(m) )\}_{1\leq i \leq m}$ attains its minimum at $i=m$. Chen et al. [6] proved that the Boros-Moll sequences are interlacing log-concave. Chen and Gu [7] showed that the sequence $\{d_{i}(m)\}_{0 \leq i \leq m}$ satisfies the reverse ultra log-concavity. Chen and Xia [8] proved that the Boros-Moll sequences are 2-log-concave, and Xia [9] studied the concavity and convexity of the Boros-Moll sequences.

In this paper, we give a new definition, i.e., skew log-concavity. Let $\{T(n,k)\}_{0\leq n < \infty, 0\leq k \leq n} $ be a triangular array of numbers. We say that $T(n,k)$ is skew log-concave if for any fixed *n*, the sequence $\{T(n+k,k)\}_{0 \leq k <\infty}$ is log-concave. We will show that the Boros-Moll sequences are almost skew log-concave.

The main results of this paper can be stated as follows.

### Theorem 1.1

*Let* $d_{i}(m)$ *be defined by* (1.4). *We have*, *for any fixed* $m\geq1$,

1.6 $$ d_{i}^{2}(m+i)>d_{i-1}(m+i-1)d_{i+1}(m+i+1), \quad i\geq1, $$

*and*

1.7 $$ d_{i}^{2}(i)< d_{i-1}(i-1)d_{i+1}(i+1), \quad i\geq1. $$

## Proof of Theorem 1.1

From (1.4), we see that $d_{m}(m)=2^{-m}{2m \choose m} $, which implies that (1.7) holds.

By (1.4),

$$d_{m}(m+1)= \frac{(2m+3)(2m+1)}{2(m+1)} 2^{-m}{2m\choose m}, $$

which yields

$$d_{i}^{2}(i+1)>d_{i-1}(i)d_{i+1}(i+2). $$

Therefore, (1.6) holds when $m=1$.

Hence, in the following, we always assume that $m\geq2$ and $i\geq1$. We first recall the following three recurrence relations derived by Kauers and Paule [3]:

2.1 $$\begin{aligned}& d_{i}(m+1)=\frac{m+i}{m+1}d_{i-1}(m)+\frac{(4m+2i+3)}{2(m+1)}d_{i}(m), \quad 0 \leq i \leq m+1, \end{aligned}$$

2.2 $$\begin{aligned}& d_{i}(m+1)=\frac{(4m-2i+3)(m+i+1)}{2(m+1)(m+1-i)}d_{i}(m) \\& \hphantom{d_{i}(m+1)={}}{}-\frac{i(i+1)}{(m+1)(m+1-i)}d_{i+1}(m), \quad 0 \leq i \leq m, \end{aligned}$$

and

2.3 $$\begin{aligned} d_{i}(m+2) =&\frac{-4i^{2}+8m^{2}+24m+19}{2(m+2-i) (m+2)}d_{i}(m+1) \\ &{} -\frac{(m+i+1)(4m+3)(4m+5)}{ 4(m+2-i)(m+1)(m+2)}d_{i}(m),\quad 0 \leq i \leq m+1. \end{aligned}$$

Now we represent the difference $d_{i}^{2}(m+i)-d_{i-1}(m+i-1)d_{i+1}(m+i+1)$ in terms of $d_{i}(m+i)$ and $d_{i}(m+i+1)$. Thanks to (2.1), (2.2) and (2.3),

2.4 $$\begin{aligned}& d_{i}^{2}(m+i)-d_{i-1}(m+i-1) d_{i+1}(m+i+1) \\& \quad = A d_{i}^{2}(m+i+1) +B d_{i}(m+i+1)d_{i}(m+i)+C d_{i}^{2}(m+i), \end{aligned}$$

where

2.5 $$\begin{aligned}& A =\frac{(4m+6i+5) (m+1+i)(m+i)(m+1)^{2}(4m+6i-1)}{ (i+1)i(4m+4i+1)(4m+4i-1)(m+2i)(m+2i-1)}, \end{aligned}$$

2.6 $$\begin{aligned}& B =-\frac{(m+1)(m+i)D}{ (i+1)i(4m+4i+1)(4m+4i-1)(m+2i)(m+2i-1)}, \end{aligned}$$

2.7 $$\begin{aligned}& C =\frac{E}{4(m+2i-1)(m+2i)(4m+4i-1) (4m+4i+1)(m+1+i)i(i+1)} \end{aligned}$$

with

2.8 $$\begin{aligned}& D= -15+400mi+35i+13m+140m^{2}+292i^{2} +864mi^{2}+688m^{2}i \\& \hphantom{D={}}{}+176m^{3}+336i^{3}+64m^{4}+72i^{4} +320m^{3}i +560m^{2}i^{2}+384mi^{3}, \end{aligned}$$

2.9 $$\begin{aligned}& E= -68mi-45i-45m-66m^{2}+2i^{2} +2\text{,}614mi^{2}+1 \text{,}901m^{2}i+451m^{3} +1\text{,}164i^{3} \\& \hphantom{E={}}{}+1\text{,}560m^{4}+3\text{,}320i^{4} +7 \text{,}732m^{3}i+14\text{,}176m^{2}i^{2}+11 \text{,}328mi^{3}+1\text{,}152i^{6} +1\text{,}984m^{5} \\& \hphantom{E={}}{}+3\text{,}392i^{5}+11\text{,}888m^{4}i +16 \text{,}856i^{4}m +27\text{,}772m^{3}i^{2}+31 \text{,}332m^{2}i^{3} +8\text{,}128m^{5}i \\& \hphantom{E={}}{}+23\text{,}040m^{4}i^{2}+9 \text{,}216i^{5}m +33\text{,}216m^{3}i^{3}+25 \text{,}216m^{2}i^{4} +6\text{,}720m^{5}i^{2}+11 \text{,}584m^{4}i^{3} \\& \hphantom{E={}}{}+1\text{,}152i^{6}m+11\text{,}072m^{3}i^{4}+5 \text{,}568m^{2}i^{5} +2\text{,}048m^{6}i +1 \text{,}152m^{6}+256m^{7}. \end{aligned}$$

It is easy to check that

$$ \Delta =B^{2}-4AC=\frac{(m+1)^{2} (m+i)F}{i(i+1)^{2}(4i+4m+1)^{2} (4i+4m-1)^{2}(2i+m)^{2}(2i+m-1)^{2}}, $$

where

$$\begin{aligned} F =& 5\text{,}184i^{8}+19\text{,}008i^{7}m+27 \text{,}648i^{6}m^{2} +19\text{,}968i^{5}m^{3}+7 \text{,}168i^{4}m^{4} +1\text{,}024i^{3}m^{5} \\ &{}+6\text{,}912i^{7}+16\text{,}128i^{6}m +768i^{5}m^{2}-33 \text{,}024i^{4}m^{3}-44\text{,}288i^{3}m^{4} -26\text{,}880i^{2}m^{5} \\ &{}-8\text{,}192im^{6}-1\text{,}024m^{7}+5\text{,}184i^{6}+13 \text{,}920i^{5}m+9\text{,}584i^{4}m^{2} -5 \text{,}936i^{3}m^{3} \\ &{}-11\text{,}648i^{2}m^{4}-5 \text{,}888im^{5}-1\text{,}024m^{6}+6\text{,}096i^{5} +23 \text{,}488i^{4}m+35\text{,}600i^{3}m^{2} \\ &{}+26 \text{,}512i^{2}m^{3} +9\text{,}728im^{4}+1 \text{,}408m^{5}+2\text{,}000i^{4}+7\text{,}232i^{3}m+9 \text{,}536i^{2}m^{2} \\ &{}+5\text{,}360im^{3}+1 \text{,}088m^{4}-1\text{,}048i^{3}-2\text{,}336i^{2}m-1\text{,}728im^{2}-404m^{3} \\ &{}-143i^{2} -175im-64m^{2}+40i+20m. \end{aligned}$$

Note that *A* is positive. Hence, in order to prove that the right-hand side of (2.4) is positive, it suffices to prove that when Δ is nonnegative,

2.10 $$ \frac{d_{i}(m+i+1)}{d_{i}(m+i)}>\frac{-B+\sqrt{\Delta}}{2A}. $$

Therefore, in the following, we assume that $\Delta\geq0$.

Recall that Kauers and Paule [3] proved the following inequality:

$$\frac{d_{i}(m+1)}{d_{i}(m)}\geq\frac{4m^{2}+7m+i+3}{2(m+1)(m+1-i)},\quad 0\leq i \leq m. $$

Replacing *m* by $m+i$, we see that

2.11 $$ \frac{d_{i}(m+i+1)}{d_{i}(m+i)}\geq \frac{4i^{2}+8im+4m^{2}+8i+7m+3}{2(m+1+i)(m+1)} , \quad i\geq0. $$

It is a routine to verify that

2.12 $$\begin{aligned}& \biggl(A \frac{4i^{2}+8im+4m^{2}+8i+7m+3}{ (m+1+i)(m+1)}+B \biggr)^{2} -\Delta \\& \quad = \frac{4(i+m)(m+1)^{2}(6i+4m+5) (6i+4m-1)G}{i(i+1)^{2}(4i+4m+1)^{2} (4i+4m-1)^{2} (2i+m)^{2}(2i+m-1)^{2}}, \end{aligned}$$

where

$$\begin{aligned} G =&28i^{4}m+108i^{3}m^{2} +144i^{2}m^{3}+80im^{4} +16m^{5}-32i^{4}-66i^{3}m \\ &{}-46i^{2}m^{2}-12im^{3}-32i^{3}-78i^{2}m -64im^{2}-17m^{3}+2i^{2}+2im+2i+m. \end{aligned}$$

Note that when $m\geq2$ and $i \geq1$, *G* is positive. Thus the right-hand side of (2.12) is positive. On the other hand,

$$\begin{aligned}& A \frac{4i^{2}+8im+4m^{2}+8i+7m+3}{(m+1+i)(m+1)}+B \\& \quad = \frac{(i+m)(m+1) (-3-12i+28im+48i^{2}+72i^{3}+32im^{2}+96i^{2}m)}{ (i+1)(4i+4m+1)(4i+4m-1)(2i+m)(2i+m-1)}, \end{aligned}$$

which is positive. Therefore, from (2.12), we have

$$A \frac{4i^{2}+8im+4m^{2}+8i+7m+3}{ (m+1+i)(m+1)}+B>\Delta, $$

which can be rewritten as

2.13 $$ \frac{4i^{2}+8im+4m^{2}+8i+7m+3}{2(m+1+i)(m+1)} >\frac{-B+\sqrt{\Delta}}{2A}. $$

From (2.11) and (2.13), we obtain (2.10) and this completes the proof.
